# Reviewing Schwannoma-Hemangioma Composite Tumors With Their Tumorigenetic Molecular Pathways and Associated Syndromic Manifestations

**DOI:** 10.7759/cureus.19839

**Published:** 2021-11-23

**Authors:** Subramaniam Ramkumar

**Affiliations:** 1 Pathology, Woodland Hospital, Shillong, IND

**Keywords:** hemangioma, merlin, vegf, tie 2, angiopoitein, hamartin, tuberin, tuberous sclerosis, schwannoma

## Abstract

Schwannomas are common peripheral nerve sheath tumors. Cavernous hemangiomas are vascular tumors that can affect any organ system. The coexistence of cavernous hemangioma with peripheral nervous system neoplasms is a rare occurrence. So far, 37 cases have been documented, and they have been divided into two categories: conjoined association (neoplasms discovered within the tumor tissue) and discrete association (neoplasms discovered outside the tumor tissue, thus placing neoplasms and tumors in close proximity but in different locations). Schwannomas and neurofibromas are the most prevalent tumors linked to cavernous hemangiomas that have been documented. The author provides a comprehensive review of all such cases published in the past with an emphasis on the implications of their tumorigenetic molecular pathways and syndromic manifestations.

## Introduction and background

The co-existence of nervous system tumors along with cavernous malformations is a rare phenomenon. Based on their location and co-occurrence, medical publications in the past have classified these lesions into two distinct types. The first type is classified as a conjoined association where the cavernous malformation is found within the tissue of the tumor. The second type is classified as a discrete association where the cavernous malformation and the tumor are in distinct separate locations in the nervous system. The conjoined association is a rare presentation with infrequent documentation of such cases in the past. The molecular aetiopathogenesis of these conjoined tumors has marked clinicopathologic implications in the therapeutic management of surgically inoperable lesions as well as in ruling out occasional associated syndromic manifestations. This review article discusses all cases published in the past as conjoined schwannoma/hemangioma tumors and the various molecular pathways implicated in tumorigenesis of such sporadic as well as syndromic tumors. Significant extrapolating facts have been derived viz these tumorigenetic molecular pathways which discusses the role of pharmacotherapy in surgically inoperable tumors. A thorough search of PubMed yielded about 70 articles with 37 previously published cases with discussions on possible tumorigenetic molecular pathways. A comprehensive compilation of the information was done which yielded four clear molecular signaling pathways which can help us in understanding and managing these rare tumors.

## Review

Discussion

The coexistence of cavernous hemangioma and nervous system neoplasms is an uncommon occurrence. There are only a few examples that have been recorded in the literature. A total of 37 instances have been reported.

History

In 1967, Willis published the first instance of concurrent schwannoma and hemangioma in the posterior mediastinum [1]. In 1917, the vascularity in neurilemmoma was explained by Cushing: “As a rule, the tumors are weakly vascularized, but in certain cases, the arteries in particular locations are sufficiently numerous to give the tumor an angiomatous look” [2]. In 1975, Moscow and Newton [3] studied the angiograms of 32 individuals with neurilemmomas of the head and neck.

They discovered that 22 (68%) of the tumors exhibited aberrant tumor vascularity; however, they did not assume that the blood vessels were angioma blood vessels. Five similar recorded occurrences involving the intraspinal, cranial nerve, and brachial plexus were described by Bojsen-Møller et al. in 1978 [4]. Kasantikul et al. described eight cases of neurilemmoma with angioma in the same lesion in 1979, and the same author described an identical lesion in the parasellar region in 1987 [5,6]. In 2006, Feiz-Erfan et al. published the first thorough analysis of cavernous malformations that are linked with neoplasms of the nervous system, including 34 examples [7]. In addition, Feiz-Efran et al. described 24 cases of conjoined associations, wherein a cavernous malformation was detected within tumors of the CNS. Out of the 19 instances, schwannoma and hemangioma were present at the same time.

Moreover, these two types of tumors can form two sorts of associations between them, and so far [7-9], 36 examples have been described. In the literature, there have been 26 cases of conjoint tumor relationships in which the tumors were detected together. There were 11 documented cases of discrete connections in which the tumors were in close proximately but were separated.

Schwannomas/neurofibromas and cavernous malformations are the most prevalent relationships mentioned above [7-9]. Schwannomas and neurofibromas accounted for 27 out of 37 instances, while gliomas accounted for the remaining 10. In our literature review, we discovered 20 examples of coexisting schwannoma with hemangioma (Table 1).

**Table 1:** Cases of conjoint association

Study	Year	Cases	Tumor type	Location of tumor
Wilson [8]	2016	1	Schwannoma	Left temporal lobe
Feiz-Erfan [7]	2006	1	Schwannoma	Cranial nerve VIII
Kogler et al. [9]	2000	1	Ganglioglioma	Occipitoparietal lobe
Acciarri et al. [10]	1994	1	Anaplastic astrocytoma	Parietal lobe
Asari et al. [11]	1992	1	Schwannoma	Cranial nerve V
Kasantikul et al. [6]	1987	1	Schwannoma	Parasellar
Chee et al. [12]	1985	1	Oligodendroglioma	Frontal lobe
Kasantikul et al. [13]	1984	1	Schwannoma	Parapharyngeal
Kasantikul et al. [14]	1982	1	Schwannoma	Cranial nerve V
Fischer et al. [15]	1982	2	Oligodendroglioma Glioma (not specified)	Frontal lobe Temporoparietal lobe
Pasquier et al. [16]	1980	1	Schwannoma	Mediastinum
Kasantikul and Netsky [5]	1979	7	Schwannoma	Ulnar nerve, Spinal cord C1-2. peroneal nerve, Cranial nerve VIII (4),
Bojsen-Moller and Spaun [4]	1978	5	Schwannoma	Intraspinal (3), cranial nerve VIII, brachial plexus
Willis [1]	1967	1	Schwannoma	Posterior mediastinum

Eight incidences were occurring in cranial nerves, and 10 were occurring in peripheral nerves. Two of the instances were intracranial, and one was intracerebral. Seven instances were included in the biggest case series documenting the conjoint tumor relationship. Based on the history, it is possible that our case is the 12th to occur in the peripheral nerves.

Five previous events that were documented occurred in patients with neurofibromatosis. In some cases, hemangioma was associated with a variety of tumors, including ganglioglioma, anaplastic astrocytoma, and oligodendroglioma [1,4,7-15,17]. Based on the literature, the most prevalent concomitant relationship with cavernous malformations is PNSTs.

A distinct connection was found in 11 cases (Table 2) [16,18-25]. Six cases had both intracranial and extracranial components; four cases had one intracranial component and one extracranial component; one case had both intracranial and extracranial components. The most prevalent tumor demonstrating a distinct relationship was neurofibroma, which was reported in five cases. The reality about these tumors is that their juxtaposition can happen by coincidence. A woman aged 47 years old [26] was diagnosed with a thoracic spine juxtaposition of extradural cavernous hemangioma and intradural schwannoma.

**Table 2:** Cases of discrete association

Study	Year	Site of cavernous malformation	Cases	Tumor type	Location of tumor
Gupta et al. [27]	2017	Right orbit	1	Schwannoma	Left orbit
Tews et al. [18]	1998	Right parieto-occipital lobe	1	Oligodendroglioma	Right parieto-occipital lobe
Mitsuhashi et al. [19]	1991	Intrasellar	1	Neurofibromatosis	Peripheral nerve
Lindboe and Nordal [20]	1985	Thoracic spinal cord	1	Neurofibromatosis	Cauda equina
Savoiardo and Passerini [21]	1978	Left occipital lobe, Right frontal lobe	2	Meningioma Astrocytoma	Left parietal lobe and right frontal lobe
Chapman et al. [22]	1959	Cerebellum,medulla oblongata	2	Neurofibromatosis	Peripheral nerve
White et al. [23]	1958	Temporal lobe	1	Astrocytoma	Septum pellucidum
Mandeville and Sahyoun [24]	1949	Fourth ventricle	1	Neurofibromatosis	Peripheral nerve
Lafora [25]	1911	Pons	1	Ependymoma	Fourth ventricle

The simultaneous emergence of two tumors with distinct etiologies may have been a coincidence. According to different recommendations, a cavernous tumor in the extradural space might be linked to altered venous outflow due to an intradural tumor or a mutation in a shared neural crest precursor cell [26]. However, the connection is usually conjoined, thus suggesting the concept of a shared molecular mechanism. In many cases, a shared genetic mechanism is a link between cavernous hemangioma and tumors of the brain.

In the tumorigenesis of concurrent schwannomas and hemangiomas, an essential role is played by the embryogenetic pathway, VEGF pathway, mTOR pathway, MAP kinase pathway, and tuberin/hamartin interactions with Rheb1/KREV1RAP1A.

Many complicated molecular cross-talk between these pathways might allow both hemangiomas and schwannomas to grow synchronously inside the same tumor. For the formation of such mixed lesions, many models have been suggested. Some writers attribute the occurrence of both neurilemmoma and angioma components to their shared ectomesenchyme origin [6]. A genetic foundation has been used in other interpretations.

Embryogenetic pathway

**Figure 1:**
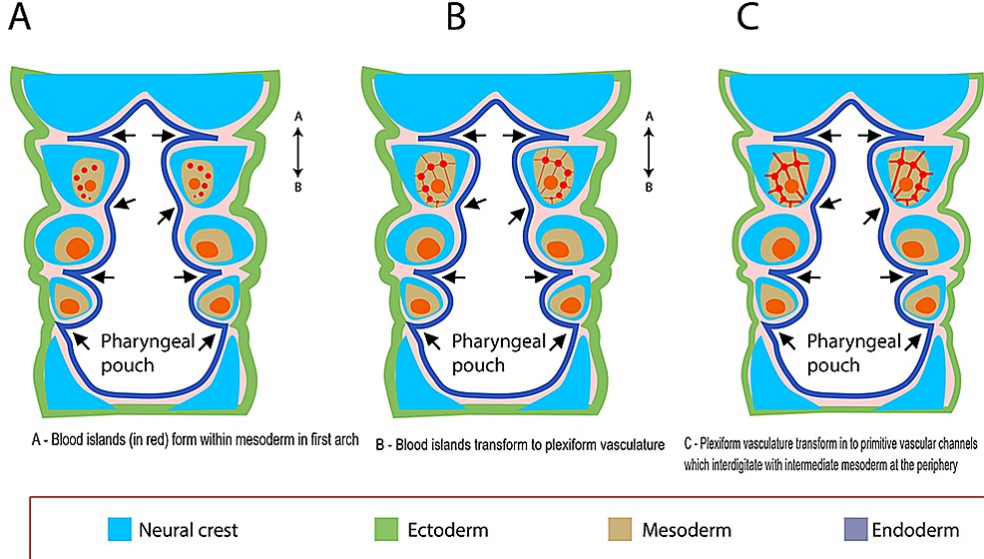
Embryogenesis and the close juxtaposition of neural crest derivatives and vascular derivatives. Copyright of the image is retained by the publishing author.

The intra-embryonic blood vessels of the human embryo begin in the first visceral arch at the late presomite stage [24]. The lateral plate becomes confined to a central core of visceral arches. The neural crest cells then migrate into the arch and contribute to forming the walls of the arch. The mesenchyme then gives rise to angiogenetic cell clusters following which they appear in the lateral plate mesoderm. Angioblasts are formed from the outermost cells of blood islands. Blood corpuscles are formed from the innermost cells or they can also disintegrate into plasma. The blood islands then form a marked plexiform structure that transforms into blood vessels [5]. Johnston and Listgarten suggested that mesodermal cells give rise to the blood vascular system, which forms capillary buds. These capillary buds extend into the surrounding mesenchyme of the neural crest. The proliferation of the neural crest cells in the arch happens in the loosely arranged lateral plate mesoderm. This leads to an intermingling of the neural crest mesenchymal cells with comparable cells of the lateral plate mesoderm [5]. Therefore, we suggest that vascular elements arise from the mesenchyme of both the neural crest and the lateral plate mesoderm. The juxtaposition of congenital cell rests of neural crest derivatives; i.e., Schwann cells and mesenchymal derivatives (i.e., the vascular system) can account for the concomitant existence of these lesions (Figure 1).

The study simultaneously analyzes similar molecular signaling pathway mechanisms operating in the neoplastic evolution of Schwann cells and endothelial cells. This helps in drawing conclusions regarding the beneficial effects of targeted therapy acting simultaneously on both components in the treatment of these hybrid lesions.

VEGF pathway

The research looks at how comparable molecular signaling pathways operate in the simultaneous neoplastic progression of Schwann cells and endothelial cells, which would support the discovery and development of targeted therapy that acts on both components concurrently in the treatment of these hybrid lesions.

VEGF/VEGFR pathway

**Figure 2:**
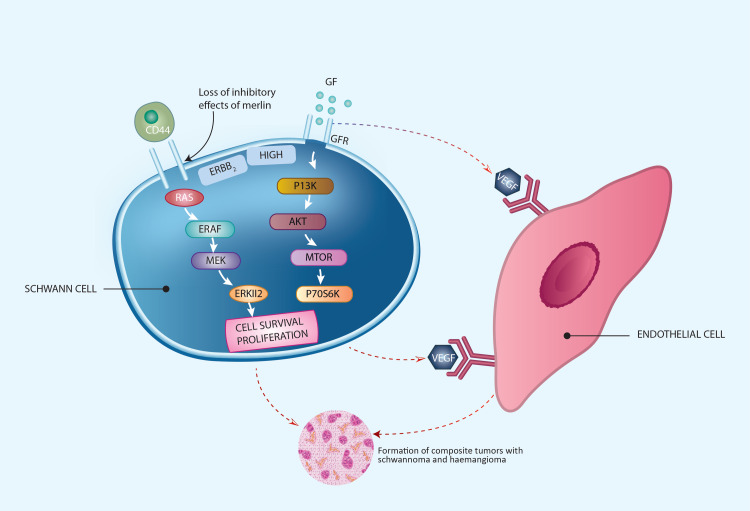
Loss of Merlin-mediated tumor-suppressive effects, MAP kinase pathway, and the PI3 kinase pathway in tumorigenesis of composite schwannoma/hemangiomas. Copyright of the image is retained by the publishing author.

In schwannomas, the loss of inhibitory effects of merlin can activate the mitogen-activated protein/ERK kinase (MEK)/extracellular signal-regulated kinase (ERK) cascade and the phosphatidylinostitol-3 kinase (PI3K)/serine-threonine protein kinase/Akt cascade, which can cause cellular growth and proliferation in schwannomas (Figure 2) [26,28]. VEGF is a key mediator of angiogenesis and is expressed in nearly all schwannomas and hemangiomas [29,30].

**Figure 3:**
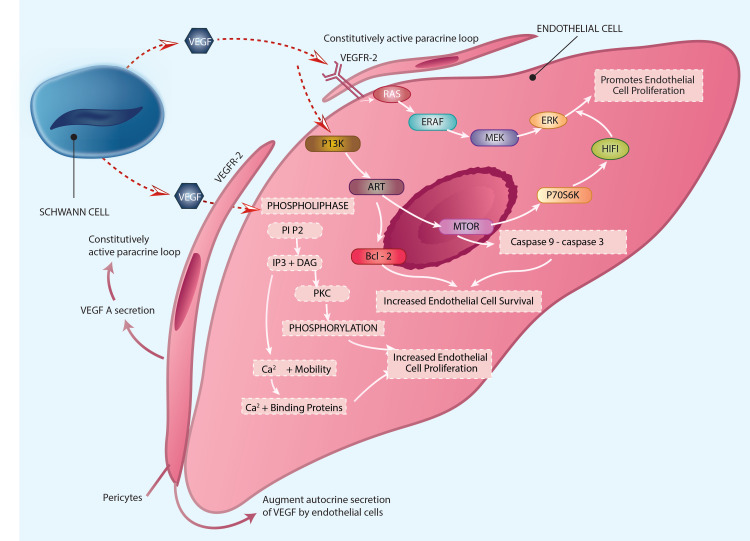
Role of VEGF/VEGFR pathway, MAP kinase pathway, PI3 kinase pathway, and the phospholipase second messenger system in tumorigenesis of hemangiomatous vasculature within a schwannoma. Copyright of the image is retained by the publishing author.

**Figure 4:**
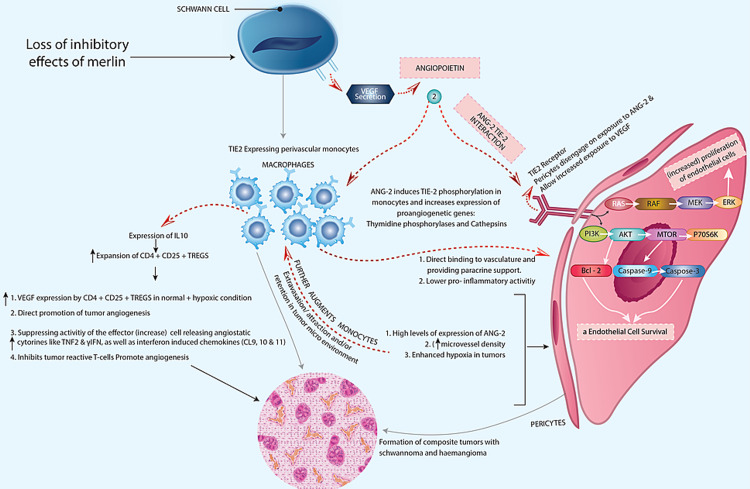
Role of angiopoietin Tie-2 interactions in the tumorigenesis of composite schwannoma/hemangiomas. Copyright of the image is retained by the publishing author.

Vasculogenesis in schwannoma and hemangioma involves binding the VEGF to its corresponding receptor [31]. Schwannomas overexpress VEGF, which increases vascular density, aberrant vascular cellular proliferation, and the schwannoma development rate (Figure 3) [30,32]. The combined and continuous growth of a hemangioma within a schwannoma can be explained by the complicated association of a schwannoma-produced VEGF with an endothelial cell (EC)/vasculature (Figures 3, 4).

Three primary secondary messenger routes are activated in ECs when the VEGF-A/VEGFR-2 complex is triggered, which results in various cascade signals that stimulate angiogenesis in a hemangioma within a schwannoma. The MEK/ERK cascade, the phosphatidylinositol-3 kinase (PI3K)/serine-threonine protein kinase/Akt cascade and the phospholipase C-/intracellular Ca2+/protein kinase C (PKC) cascade are the three pathways involved. Subsequent angiogenic effects of VEGF-A, such as microvascular permeability, EC proliferation, migration, and survival, are then activated [27,33,34]. This might possibly play a part in EC adaptation in a tumor setting, as well as shielding ECs versus apoptotic cell death (Figure 3) [35]. The VEGF-A/VEGFR-2 interaction also creates a persistently autocrine loop in EC cells and makes Hem EC cells more sensitive to paracrine activation [36]. This leads to enhanced cell proliferation and tumor development. Pericytes can increase the autocrine production of VEGF by tumor ECs and create VEGF-A in an autocrine way, which can have a paracrine impact (Figure 3) [37,38].

Finally, COSMC has been associated with a significant increase in developing hemangiomas, which has been linked to increased VEGF-mediated phosphorylation of VEGFR-2 and downstream signaling [39]. The survival of ECs may benefit from the aberrant activation of VEGFR-2 on the cell surface.

As a result, the absence of Merlin's inhibitory activity and the stimulation of VEGF-A with VEGFR-2 can enhance transmission in proliferating Schwann cells/ECs, thus promoting the formation of a hemangiomatous vasculature inside a schwannoma.

Elucidating the molecular cross-talk in hemangiomas and schwannomas can lead to the development of combination targeted therapy for these uncommon composite cancers. In a clinical trial of bevacizumab [40], 31 individuals with NF2-associated vestibular schwannomas and hearing loss were studied. Auditory enhancement was shown in more than half of the patients; however, the therapeutic effect required long-term dosage, which could lead to chronic systemic toxicity. Dose adjustment regimens have been used in studies aiming to lessen the negative impacts of bevacizumab.

The amount of VEGF circulating in the blood can be used as a serum indicator to determine which people may benefit the most from anti-VEGF medication. As a result, the abundance of VEGF generated by schwannoma cells was linked to the tumor development rate [41].

According to multiple preclinical investigations, targeting mitogen-activated protein kinase (MAPK) signaling, such as with the application of MEK inhibitors, is another approved treatment that can address both the Schwann cell and vascular component of hybrid composite tumors, making this approach worthy of further exploration [42,43].

mTOR pathway

The mTOR pathway has been established as one of the most essential routes controlling the proliferation of cells. The overactivation of the PI3K/Akt/mTOR pathway, a signaling pathway that plays a key role in cellular growth and survival, has been implicated in various tumor pathogeneses [44,45]. The binding of corresponding growth factors to their receptors stimulates the phosphatidylinostitol-3 kinase (PI3K)/serine-threonine protein kinase/Akt cascade. PI3K generates 3-phosphorylated inositol lipids, which causes the activation of downstream signaling, thus resulting in the activation of protein kinase B (PKB; also called c-Akt), which regulates mTOR, glycogen synthase kinase-3β, and Forkhead box O transcription factor activities, among others. Downstream targets of mTOR include p70 ribosomal protein 6S kinase (S6K) (Figures 2, 3) [29]. The above sequential cascade results in increased cell survival and cell proliferation.

Merlin-mediated mTOR signaling suppression has been demonstrated to assist Merlin's tumor suppressive action in schwannomas (Figure 2) [44]. In hemangiomas, the binding of VEGF-A with VEGFR-2 stimulates the mTOR pathway resulting in increased cell proliferation and survival of endothelial cells (Figure 3).

As a result, the lack of Merlin-mediated regulation of mTOR signaling in a schwannoma, as well as the VEGF-A/VEGFR-2 enhanced PI3K/Akt/mTOR pathway in ECs inside a schwannoma, might result in synchronous hemangiomatous development. Hence, the suppression of the PI3K/Akt/mTOR pathway is also of therapeutic interest [46-48].

Pharmacological suppression of mTOR signaling can diminish schwannoma development in vivo, according to allograft research in mice [49]. Studies on the self-renewal capability of HemSCs in vivo and in vitro studies have shown that rapamycin (mTOR inhibitor) can impair or decrease their capacity to differentiate and block their vasculogenic activity in vivo [48]. Therefore, mTOR inhibitors would be extremely beneficial in the therapy of these hybrid lesions.

Angiopoitein-Tie-2 interactions

The development and advancement of schwannomas are determined by the degree of macrophage function. The number of cd68-positive cells, tumor size, tumor growth index, and increase in tumor microvessel density have a clear correlation with the vascular density of a schwannoma [50]. Tie-2 and angiopoietins (Ang) form a receptor-ligand system that has a role in both physiological and pathological processes. The Tie-2 tyrosine kinase receptor is found in a variety of macrophages that are involved in angiogenesis and vascular ECs [51]. Yu et al. found that Tie-2 was upregulated in HemECs and that this upregulation was linked to an improvement in cellular signaling to the Tie-2 agonist Ang-1 [52].

Higher expression of proangiogenic genes such as thymidine phosphorylase and cathepsins is caused by Ang-1-mediated Tie-2 connections on monocytes. Furthermore, Ang-Tie-2-activated macrophages produce more IL-10, which causes regulatory T cells to release more VEGF and less angiostatic cytokines [53,54]. Activated macrophages by Ang-Tie-2 bind to activated ECs, which provides paracrine assistance and reduces pro-inflammatory action. Pericytes disengage as a result of Ang-2 Tie-1 interactions, which enables greater VEGF exposure.

This activates and stimulates a variety of intracellular signaling pathways, including the PI3K/Akt pathway and MAPK pathway [53-55], which increases the EC lifespan. As a result, the existence of a greater number of CD68 histiocytes in a schwannoma can cause angiopoietin-Tie-2 interactions and consecutive extensive vascular proliferation (Figure 4) [53].

Various ways to control macrophage activation are now being researched [56,57], making a thorough analysis of the inflammatory process in vestibular schwannomas even more intriguing. In tumors with a large number of CD68-positive cells, the microvessel density was considerably greater [50]. Hemosiderin deposition was shown to be considerably higher in cystic and inhomogeneous tumors than in homogenous tumors [58,59]. The amount of intratumoral inflammation (which is mostly determined by CD68 histiocytes); tumoral bleeding; tumoral hemosiderin deposition; vascularization; and microvessel thickness all play a role in the growth of vestibular schwannomas.

As a result, more research into the types of inflammatory cells in schwannomas, their activation states, and their relationships with angiogenic growth factors will further elucidate the underlying molecular mechanisms, which will lead to the discovery of remedies that may be used to treat them.

KRIT1 gene Krev-1rap1a pathway

**Figure 5:**
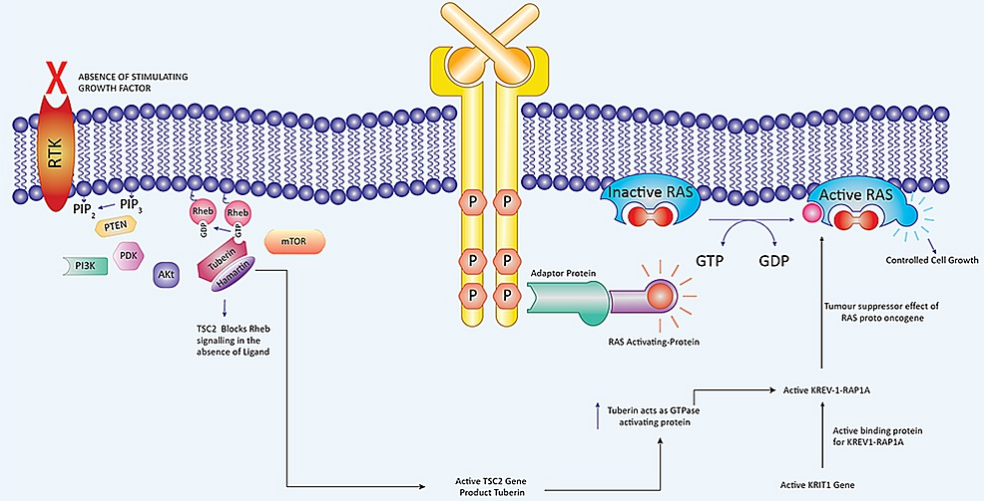
Tumor suppressive effects of tuberin-Hamartin interactions, KRIT1 gene Krev-1rap1a pathway, and RAS proto-oncogene. Copyright of the image is retained by the publishing author.

**Figure 6:**
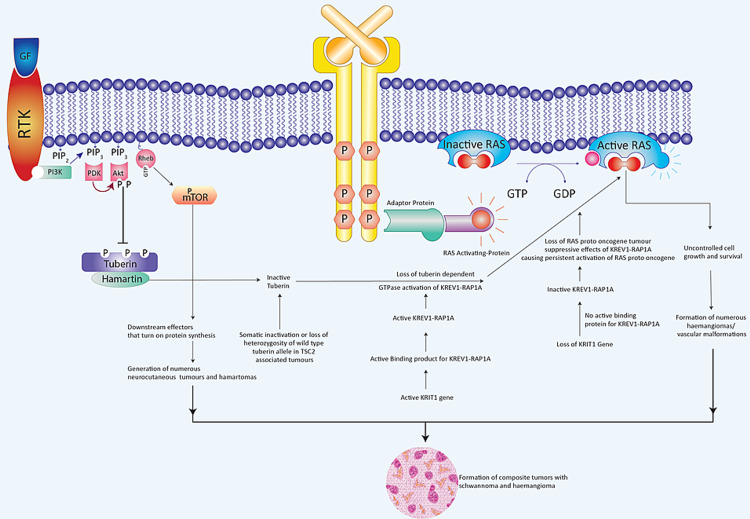
Role of tuberin-hamartin interactions, KRIT1 gene Krev-1rap1a pathway, and RAS proto-oncogene in tumorigenesis of composite schwannoma and hemangiomas. Copyright of the image is retained by the publishing author.

Past scientific studies have demonstrated a potential hereditary path that might be accountable for the coexistence of neurocutaneous tumors and hemangiomas. This has been seen in tuberous sclerosis, where the tuberin/hamartin complex loses its inhibitory impact on Rheb signaling while losing its stimulatory impact on the guanosine triphosphatase (GTPase)-activating protein for Krev-1=rap1a. As a result of the former, multiple neurocutaneous tumors and hamartomas are produced. As a result of the latter, numerous cerebral cavernous vascular malformations/hemangiomas are formed (Figures 5, 6) [7,60].

Signaling Pathway in the Generation of Multiple Neurocutaneous Tumors in Tuberous Sclerosis

Tuberin is a protein that is encoded by the TSC2 gene. Rheb is a small GTPase molecule that stimulates the mTOR/S6 kinase (S6K)/factor 4E binding protein (4E-BP)-1 signaling cascade, which is critical for cell size and proliferation control and is negatively regulated by the tuberin-hamartin complex. The tuberin-hamartin complex might also influence the development of the cyclin-dependent kinase inhibitor p27Kip1, which is important in the development and size regulation of cells [61].

In patients with tuberous sclerosis, the tuberin-hamartin complex-mediated inhibition of cell size and proliferation is relieved by the activation of phosphatidylinositol 3-kinase (PI3k) and Akt after stimulation of the insulin-like growth factor receptor. The Akt phosphorylates tuberin, thereby inactivating the complex, which increases cell size and proliferation. This results in the generation of multiple neurocutaneous tumors [7,61].

Signaling Pathways in the Generation of Hemangiomas in Tuberous Sclerosis

Three distinct loci for genetic mutations that are associated with cerebral cavernous malformations have been identified so far. CCM1 on the long arm (7q21-22) and CCM2 on the short arm (7p15-13) of chromosome 7, and CCM3 on the short arm (3q25-27) of chromosome 3 [7]. The affected KRIT1 gene is situated on the long arm of chromosome 7 (7q21-22). This gene codes for a binding protein that is associated with Krev-1=rap1a [62,63]. Krev-1=rap1a in turn functions as a tumor-suppressor protein by binding to a number of the effectors of the Ras oncogene (Figures 5, 6) [64,65].

The loss of KRIT1 function, which triggers the tumor inhibitory effects of Krev-1-rap1a, would further boost Ras protooncogene effects. As a result of the lack of Krev-1rap1a's tumor-suppressing actions, people with mutations in the KRTI1 gene are more likely to develop cavernous malformations. This idea is supported by the observation of a modification of the Krev-1=rap1a pathway in tuberous sclerosis type 2 (TSC2), which is an autosomal dominant disorder indicated by benign neurocutaneous tumors.

Tuberin, which is a tumor-suppressor protein produced by the TSC2 gene, acts as a guanosine triphosphatase (GTPase)-activating protein for Krev-1=rap1a [63]. Somatic inactivation or loss of heterozygosity of the wild-type tuberin allele in TSC2-associated tumors leads to constitutive activation of Krev-1/rap1a and unregulated growth [21,66]. This results in the generation of cerebral cavernous vascular malformations. The loss of the wild-type KRIT1 allele can result in the focal nature of CCMs during the development of cerebral vasculature. A two-hit model, which has a central tumor-suppressor function of KRIT1, would be similar to the mechanism of tuberin inactivation in TSC2-associated tumors [7].

Furthermore, we can extrapolate the above concept to a common tumorigenetic molecular cross-talk pathway between PNSTs and CCVMs. The effects of tuberin on Rheb signaling and the GTPase activating profile on KREV1RAP1a may be responsible for the concomitant development of neurocutaneous tumors (e.g., schwannomas) and vascular lesions (cerebral cavernous vascular malformations).

In fetal or neonatal rat brains, oncogenes given using retroviral vectors can cause tumors (gliomas) and cavernous malformations, which indicates a common genetic flaw as a possible etiology [67,68]. The co-occurrence of cavernous malformations with both gliomas and tumors of Schwann cell origin may be explained by the fact that chromosome 7 monosomy and 7q22 deletions occur as common secondary events after hyperactivation of Ras oncogenes or mutation of the Ras-GAP protein neurofibromatosis-1 (NF-1) gene [68,69].

The research has indicated a significant risk of bleeding in patients of vestibular schwannoma coupled with cavernous hemangioma. This result is linked to the “vascular instability” that is caused by higher levels of matrix metalloproteinase-2 and -9, which are hypothesized to be induced by tumor cytokines. The aberrant arteries are prone to thrombosis and bleeding [16,70].

Even if such complex lesions arise in the CNS, hemorrhages, particularly subarachnoid hemorrhages, have also been recorded. Vascular malformations and PNSTs have similar characteristics upon imaging, which can cause the radiologist to mistake one for the other. PNSTs can be misinterpreted for vascular malformations because they are hypointense on T1-weighted imagining and hyperintense on T2-weighted imaging; these features improve with greater contrast [70].

## Conclusions

The association of cavernous malformations/hemangiomas with schwannomas might be related to the complex cross-talk between the common tumorigenetic signal pathways that are involved in schwannomas and hemangiomas. The pathways implicated in schwannoma tumorigenesis are the loss of Merlin-mediated inhibition of the MAP kinase MEK/ERK cascade), and the PI3K/mTOR signaling pathways (phosphatidylinostitol-3 kinase (PI3K)/serine-threonine protein kinase/Akt cascade/mTOR/p70S6K). The pathways implicated in hemangioma tumorigenesis are the MAP kinase pathway MEK/ERK cascade, PI3 kinase pathway (phosphatidylinostitol-3 kinase (PI3K)/serine-threonine protein kinase/Akt/mTOR/p70S6K cascade), and the phospholipase second messenger system (phospholipase C-γ/intracellular Ca2+/(protein kinase C (PKC) cascade). Schwannoma-secreted VEGF and angiopoietin-Tie-2 interactions act as the prime modulator in triggering and self-perpetuating the above-described complex signaling interactions between Schwann cells and ECs, which results in concomitant growth of schwannoma and hemangioma in the same tumor.

Furthermore, previous data suggest that inherited and genetic implications play a role in the development of these composite tumors, as was observed in patients with tuberous sclerosis. There is a concomitant loss of the suppressive effect of the tuberin/hamartin complex on Rheb signaling and the loss of the stimulatory effect of tuberin on the guanosine triphosphatase (GTPase)-activating protein for Krev-1=rap1a. The former results in the generation of neurocutaneous tumors, and the latter results in the generation of concomitant cavernous vascular malformations/hemangiomas in the same patient. The presence of this particular variant of composite tumor in a patient implies that genetic screening and genetic counseling for possible tuberous sclerosis should be performed in susceptible patients. A subtle expression of the disease can happen due to the variable penetrance of the associated mutations. That chromosome 7 monosomy and 7q22 deletions occur as common secondary events after hyperactivation of Ras oncogenes or mutation of the Ras-GAP protein neurofibromatosis-1 (NF-1) gene may explain the co-occurrence of cavernous malformations with both gliomas and tumors of Schwann cell origin.

The documentation of 21 cases of concomitant schwannoma and hemangioma mandates the inclusion of an “Angiomatous variant of Schwannoma” under the World Health Organization classification of morphologic subtypes of schwannomas. Furthermore, the molecular cross-talk pathways and previously documented data imply that surgically inoperable schwannomas with high vascularity and/or a hemangiomatous component may benefit from targeted therapy with drugs like MEK inhibitors, mTOR inhibitors (rapamycin), NSAIDs (e.g., aspirin), and bevacizumab (VEGF monoclonal antibody).
